# CD96 as a Potential Immune Regulator in Cancers

**DOI:** 10.3390/ijms24021303

**Published:** 2023-01-09

**Authors:** Shikai Feng, Orkhan Isayev, Jens Werner, Alexandr V. Bazhin

**Affiliations:** 1 Department of General, Visceral and Transplant Surgery, Ludwig-Maximilians-University Munich, 81377 Munich, Germany; 2 Department of Cytology, Embryology and Histology, Azerbaijan Medical University, Baku AZ1022, Azerbaijan; 3 German Cancer Consortium (DKTK), Partner Site Munich, 81377 Munich, Germany; 4 Bavarian Cancer Research Center (BZKF), 91054 Erlangen, Germany

**Keywords:** CD96, CD155, CD226, immune checkpoint molecules, immune modulation, TIGIT

## Abstract

The discovery of CTLA-4 and PD-1 checkpoints has prompted scientific researchers and the pharmaceutical industry to develop and conduct extensive research on tumor-specific inhibitors. As a result, the list of potential immune checkpoint molecules is growing over time. Receptors for nectin and nectin-like proteins have recently emerged as promising targets for cancer immunotherapy. Potential immune checkpoints, including CD226, TIGIT, and CD96, belong to this receptor class. Among them, CD96 has received little attention. In this mini-review, we aim to discuss the basic biology of CD96 as well as the most recent relevant research on this as a promising candidate for cancer immunotherapy.

## 1. Introduction

Malignant tumors are one of the most prominent issues the world is currently dealing with, which is at the top of the lists of all lethal factors that result in fatalities each year. Currently available therapies for tumors include surgical resection, radiotherapy, and chemo- and immune therapy [1,2]. However, the biological characteristics of tumor cells, such as rapid growth, metastasis, and resistance to chemoradiotherapy, make it challenging to completely remove tumor cells through conventional treatment programs, ultimately resulting in tumor recurrence [3,4]. Although these treatments can effectively kill tumor cells, they can also be extremely harmful to patients. Moreover, radiotherapy, chemotherapy, and other treatments kill normal cells, as they are not highly targeted. In recent years, the use of immune checkpoint blockade (ICB) has made great progress in cancer treatment. The first immune checkpoint inhibitor, ipilimumab, was approved by the US FDA in 2011 for the treatment of advanced melanoma [5]; in 2018, Professor James Allison, an immunologist at the University of Texas, and Professor Tasuku Honjo, a professor at Kyoto University, were awarded the Nobel Prize for research on immune checkpoint inhibitors [6]. Despite considerable progress of immune checkpoint blockade therapies, a number of issues still exist, including low responsiveness, adverse effects, and drug resistance [7]. In the context of low responsiveness, the combination of immune checkpoint blocking agents is anticipated to improve response rates and patient survival. Currently, there are more drugs targeting the immune checkpoints PD-1 and CTLA-4. A global phase III clinical trial showed that the combination of PD-1 and CTLA-4 for advanced melanoma had a response rate of 58%, a complete response rate of 11.5%, and significantly prolonged progression-free survival [8,9,10]. Additionally, it has been found that using immune checkpoint inhibitors in combination with radiotherapy or chemotherapy can enhance patient outcomes. Therefore, uncovering immune checkpoints that are highly effective could help improve the efficacy of immune checkpoint blockade therapies. Recently, attention has been drawn to receptors for nectin and nectin-like proteins as promising targets for cancer immunotherapy, including potential immune checkpoints as CD226, TIGIT, and CD96. Among them, CD96 has received relatively little attention. In this review, we discuss the basic biology of CD96 as well as the most recent relevant studies.

## 2. Molecular Structure of CD96

**Figure 1:**
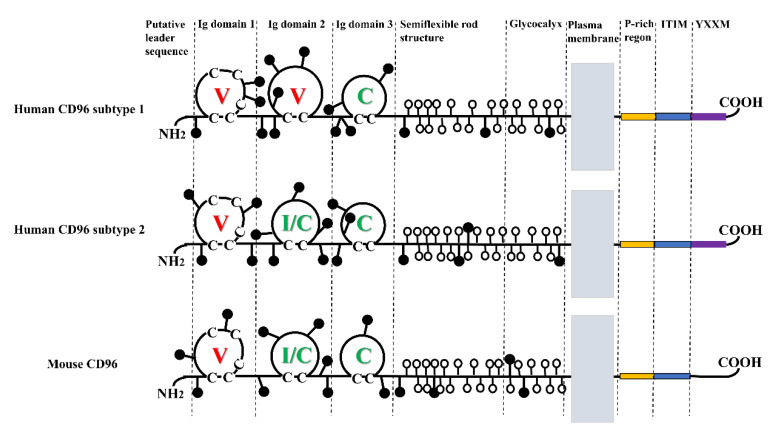
Molecular structure of CD96. CD96 has three Ig domains. The domain of hCD96 subtype1 is V, V, and C; the domain of hCD96 subtype2 and mCD96 is V, I/C, and C. Where the c on the domain loop refers to cysteine. N-glycosylation sites indicated by attached black circles; serines and threonines in the putative O-glycosylated region are indicated by attached open circles.

The immunoglobulin superfamily (IgSF) was first introduced in the 1980s. A majority of IgSF molecules are predominantly transmembrane glycoproteins and serve as cell adhesion molecules [11]. PL Wang et al. discovered CD96, an IgSF member also known as TACTILE in 1992 [12]. According to SDS-PAGE experiments, CD96 is 160 kDa in size in the reducing state, while it can be 160, 180, and 240 kDa in the nonreducing state, but 180 kDa is not common [12]. The 1.5 kb long nucleotide sequence for CD96 has an ATG beginning nucleotide sequence and a 928 bp non-coding region, which is followed by a 2.5 kb non-coding region [12]. The molecular structure of CD96 is depicted in Figure 1. 

CD96 consists of an extracellular structural domain, flexible neck domain, transmembrane structural domain, and cytoplasmic structural domain [13]. Based on the number of residues between cysteines, the extracellular structural domains of immunoglobulins can be divided into three subtypes: the V structural domain (65–75 residues), the C structural domain (55–60 residues), and the H structural domain (35–55 residues) [12]. Human CD96 consists of three structural domains. It undergoes selective splicing in the second structural domain to produce two isoforms with distinct Ig folding [14]. Subtype 1 consists of 568 amino acids extracellularly, and its structural domains are V, V, and C. In subtype 2, the second Ig structural domain lacks the 16 amino acids encoded by exon 4, forming a type I or C structural domain with V, I/C, and C [14]. The second isoform of human CD96 is comparable to the three structural domains found in mouse CD96 [13]. The flexible neck region consists of stretch rich in serine/threonine/proline, which is highly glycosylated with numerous O-linked disaccharides and present adjacent to the transmembrane region and has a rod-like structure [15]. The structural domain of CD96′s transmembrane domain is shared with other IgSF as well. The cytoplasmic structural domain of CD96 consists of 45 amino acids and is composed of P-rich region, an immunoreceptor tyrosine-based inhibitory motif (ITIM), and the YXXM motif. Notably, the YXXM motif is absent from mouse CD96 [13].

## 3. CD155 as the Main Ligand of the CD96 Receptor 

**Figure 2:**
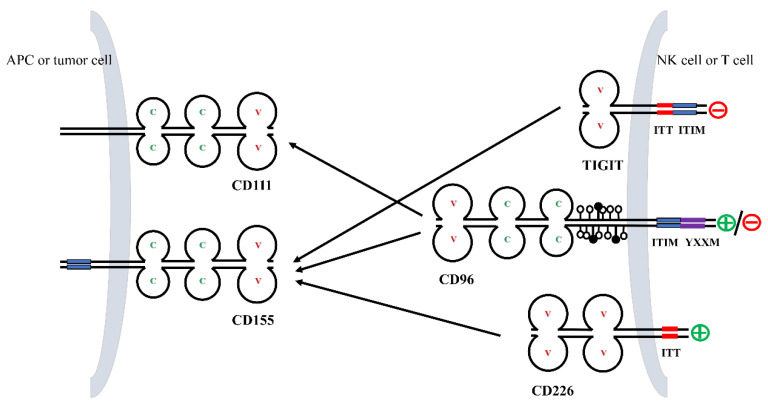
Interactions between members of the CD96 axis. TIGIT, CD96, and CD226 on NK cells and T cells bind to their ligands CD155 and CD111 with different affinities. Residues in the cytoplasmic tail of TIGIT, CD96, and CD226 are phosphorylated and generate co-stimulatory or co-inhibitory signals.

CD155 serves as the primary ligand for CD96 (Figure 2). It can interact with and activate receptors, like TIGIT and CD226, to exert immunomodulatory effects [13] (Figure 2).

The immunoglobulin superfamily’s conserved amino acid and structural domain profile shared by CD155 is considered to be a receptor for polio virus [16,17]. CD155, also known as necl-5, is a member of the nectin-like molecule family, which includes four nectins (nectin1-4) and five necls (necl1-5), and its molecular domain is similar to that of nectin, which is involved in cell adhesion and polarization [18,19,20].

TIGIT is a receptor mainly expressed by T cells and NK cells that inhibits immunoglobulins. TIGIT contains a variable extracellular immunoglobulin structural domain, a type I transmembrane structural domain, and a short intracellular structural domain that contains an immunoreceptor complexine-based inhibitory motif (ITIM) and an immunoglobulin complexine tail (ITT)-like motif [21,22,23]. Although CD96 has a role similar to that of TIGIT, the binding affinity of CD96 (37.6 nmol/L) to CD155 is comparatively lower than TIGIT (1–3 nmol/L).

CD226, also known as DNAM-1 or nectin-2, is expressed in NK cells, T cells, B cells, monocytes, macrophages, dendritic cells, and other cells [24,25,26,27]. As an activating receptor, CD226 competes with TIGIT and CD96 for its binding to CD155, but with lesser affinity (114–199 nmol/L) when compared to TIGIT and CD96 [15]. 

A short, an amino acid-rich sequence in an ITIM-like domain in the mouse and human cytoplasmic structural domains suggests CD96 as a potential immune cell suppressor [15]. A vast majority of studies have shown that inhibition of the immune checkpoint CD96 can be used as a therapeutic approach to enhance immune cell activity and inhibit tumor growth [28,29]. The presence of the YXXM motif in the cytoplasmic structural domain of human CD96 suggests that CD96 may have the ability to function as an activating receptor under certain conditions [30]. 

TIGIT inhibits NK cells and T lymphocytes, whereas CD226, an activating receptor, can kill CD8^+^ T lymphocytes without the need for other co-stimulatory factors [31,32,33]. Co-inhibition of TIGIT and CD96 and co-stimulation of CD226 is analogous to the CTLA-4/CD28 pathway. In addition, the expression levels of TIGIT, CD96, and CD226 were similar to those of CTLA-4/CD28, and in response, the co-stimulatory receptors were expressed on naive and resting T cells, whereas co-inhibitory receptors were expressed upon T cell activation [21,34]. Only the outermost V-type extracellular structural domain of CD96 can bind to CD155, and the second and third structural domains can influence the interaction [14]. In addition, some studies have reported a weak and low-affinity bond between CD96 and nectin-2 [35]. Mouse CD96 has also been shown to bind to nectin-1 [14,36].

## 4. Cellular and Tissue Distribution of CD96

The enhanced expression of CD96 at later stages after T cell activation led to its discovery [12]. In humans and mice, CD96 is predominantly expressed in T cells and CD56^bright^ NK cells, but with little or no expression in B cells, monocytes, δ T cells, and Treg cells.

T cells are broadly classified into naive T cells, effector T cells, either cytotoxic T cells (CD8^+^) or helper T cells (CD4^+^), and memory T cells subtypes: effector or central memory T cells. Studies report that CD96 is strongly expressed on both effector memory and central memory CD8^+^ T cells, respectively, as well as on CD4^+^ T effector memory cells. Among the CD4^+^ T cell subtypes (Th1, Th2, and Th17), CD96 was highly expressed in Th1 cells [37]. At the transcriptional level, CD96 is highly expressed in T cells and NK cells; moderately expressed in lymph nodes, tonsils, bladder, spleen, appendix, and B cells; and lowly expressed in other tissues, such as small intestine, granulocytes, and bone marrow (Table 1). Immunohistochemistry (IHC) stains showed that CD96 protein expression was mainly distributed in the cytoplasm and cell membrane [38]. This distribution of CD96 genes in cancer tissues showed low cancer specificity, and all cancer tissues had very low levels of CD96 gene expression, with the stroma of cancerous tissues expressing moderately [38]. However, various databases show different levels of CD96 gene expression in tumor and normal tissues. 

The Oncomine database and TCGA database are two powerful databases for searching for cancer gene information. Using these two databases, we can better understand the relationship between genes and cancer [39,40]. For instance, in the Oncomine database, CD96 mRNA expression was found to be higher in cancer tissues from brain, breast, kidney, and leukemia than in paracancerous tissues. Likewise, in the TCGA database, the CD96 gene was shown to be expressed higher in adrenocortical cancer, breast invasive cancer, endocervical adenocarcinoma, cholangiocarcinoma, colon adenocarcinoma, esophageal carcinoma, glioblastoma multiforme, head and neck squamous cell carcinoma, kidney renal clear cell carcinoma, kidney renal papillary cell carcinoma, acute myeloid leukemia, lower grade glioma, liver hepatocellular carcinoma, ovarian serous cystadenocarcinoma, pancreatic adenocarcinoma, skin cutaneous melanoma, stomach adenocarcinoma and testicular germ cell tumors compared to normal tissue. On the contrary, CD96 mRNA levels were lower in cancers of lung, rectal, and thyroid compared to normal tissue [38] (Table 1). 

To investigate whether CD96 is involved in the immune infiltration process of pan-cancer, the ESTIMATE algorithm can be used to evaluate the proportion of stromal cells in tumor microenvironment (TME), the proportion of immune cells in TME and the combined proportion of the above two cells in TME in each sample, using the Stromalscore, Immunescore, and Estimatescore algorithms, respectively. These findings demonstrate that CD96 abundance significantly correlates with the Stromalscore of colon adenocarcinoma, glioblastoma multiforme, and head and neck squamous cell carcinoma. In contrast, CD96 abundance significantly correlates with the Immunescore of invasive breast cancer, cervical canal adenocarcinoma, and bile duct carcinoma. In endocervical adenocarcinoma, colon adenocarcinoma, and esophageal cancer, the Estimatescores strongly correlates with CD96 abundance. Thus, these results indicates that CD96 is involved in the immune infiltration process in the above-mentioned tumors [38]. Next, to study the correlation between CD96 and pan-cancer infiltration of various immune cells, the IMER 2.0 database was used. The results from IMER 2.0 demonstrated a positive correlation of CD96 with the infiltration levels of CD8+ T cells, dendritic cells, monocytes, NK cells, neutrophils, regulatory T cells, and follicular helper T cells. However, a negative correlation between CD96 and infiltration levels of myeloid-derived suppressor cells was observed. Overall, we can speculate that CD96 might be involved in the immune infiltration process of majority of immune cells [38].

It is important to note that there are currently no data on CD96 expression in T-cell lymphoma, which opens up new avenues for investigation of CD96 in in relation to this malignancy. 

## 5. Functions of CD96

CD96 is relatively a new molecule. Functions of this receptor have not yet been fully investigated and understood. However, involvement in inflammation and molecular adhesion are currently well-known functions. 

### 5.1. Anti-Inflammation

T helper type 9 (Th9) cells can be divided into two subtypes: CD96^high^ and CD96^low.^ The CD96^low^ Th9 cells exhibit pro-inflammatory functions, while CD96^high^ Th9 cells do not. It has been shown that CD96^low^ Th9 cells have higher levels of IL-9 mRNA and protein, as well as the potential for IL-4 and IL-5 expression. In addition, it is shown that blocking CD96 could make theCD96^high^ Th9 cells lose their anti-inflammatory properties [41]. Overall, this suggests CD96 has a potential anti-inflammatory property.

### 5.2. Mediates the Adhesion Function

Studies have shown that CD96 mediates the adhesion of NK cells to cells expressing the myeloid virus receptor (PVR)/CD155, thereby facilitating their interaction and thus stimulating the cytotoxic activity of NK cells [42]. This pro-adhesive property of CD96 and a higher expression of PVR in certain tumors aids in tumor recognition and tumor cell death by NK cells.

## 6. Mechanisms of CD96 Regulation of Tumor Immunity

CD96 regulates the activities of NK cells and CD8^+^ T cells and is an important factor in regulating tumor immune function (Figure 2). 

### 6.1. Controlling and Regulation of NK Cells

Human natural killer (NK) cells are innate lymphocytes that play a crucial role in the defense against virus infected cells and tumors, accounting for 15% of all circulating lymphocytes [43]. They can be divided into two main subpopulations: CD56^bright^CD16^−^ and CD56^dim^CD16^+^. The CD56^bright^CD16^−^ is generally considered less mature and a potent cytokine producer, while the latter is more mature and cytotoxic [44,45]. Most NK cells in the blood are CD56^dim^ and less than 15% are CD56^bright^ [44]. They have two distinct functional cell surface receptors that distinguish normal cells from virus-infected or malignant cells. A specific NK cell receptor targets for cell surface molecules that are highly expressed in viral infected and tumor cells but not on the normal cell surfaces, thereby stimulating NK cell cytotoxic activity. CD226 is one of such receptor, which can stimulate the killing of NK cells by controlling interferon production against various tumors and viral infected cells. Another type of NK cell receptor detects cell surface molecules expressed on normal cells only and transmits inhibitory signals that protect them from invasion by the NK cell. Most of the known NK cell inhibitory receptors are specific for classical or non-classical MHC class I molecules expressed by normal cells [18]. 

Malignant cells have evolved in multiple ways to evade recognition and killing by the immune system. Tumor cells, for example, downregulate antigen presentation receptors or inhibit the recognition by immune cells; tumor cells can also create an immunosuppressive microenvironment by recruiting myeloid cells, regulatory T cells, and other regulatory cells to secrete suppressive cytokines such as IL-10 and TGF-β. Additionally, tumor cells activate immune checkpoints, thereby inhibiting the effector function of NK cells [46]. CD155, an immunoglobulin adhesion molecule, is not or lowly expressed in normal human tissues, while it is primarily overexpressed in human malignancies. As a result, the expression of co-stimulatory receptors CD226 and co-inhibitory receptors TIGIT and CD96 on NK cells suggest that CD155 may play a dual role in tumor immunity [47]. Even though CD96 was discovered 20 years ago, its function is has not been completely unraveled especially related to immune response in cancer. 

Human CD96 and mouse CD96 have distinct structures. The ITIM structural domain is found in both human and mouse cytoplasmic tails, but only human cytoplasmic tails contain the YXXM motif [13]. Following the discovery of the immune receptor tyrosine activation motif (ITAM), the concept of ITIM structural domain was introduced in 1995. ITAM was originally identified as a member of the low-affinity immune Globulin G (IgG) receptor family. Experimental results showed that binding of IgG immune complexes to B cell receptors inhibits B cell activation [48]. Molecular analysis of the intracytoplasmic sequence of FcγRIIB revealed the presence of a complex at the Y+3 site where leucine follows, and point mutations in this complex could eliminate FcγRIIB inhibitory properties. 

Subsequently, receptors of major histocompatibility complex class I (MHC-I) molecules were identified in NK cells. The intracytoplasmic structural region involved in “ITAM-like” sequences with 2 Yxxl motifs separated by 26 amino acids in killer Ig superfamily receptors (KIR), which are unlikely to contain two SH2 structural regions of Syk kinase, which have been shown to require two complexes at specific spacing for their recruitment and were hypothesized to possibly correspond to two tandem ITIMs, with studies confirming this conjecture [49]. Moreover, upon co-aggregation with the high-affinity IgE receptor (FceRI) expressed by these cells, both could inhibit IgE-induced mediated release, phosphorylated by complex amides, and also recruit SH2 structural region-containing cytoplasmic phosphatases. Besides, studies show that Fcγ-type receptors interacted with various phosphatases. For example, Fcγ RIIB collected a single SH2 structural region-containing phosphatidylinositol 5-phosphatase (SHIP), while the FcγRIIB-KIR chimera collected a two SH2 structural region-containing complex phosphatase (SHP). Based on this, the concept of ITIM-mediated negative feedback regulation quickly gained popularity [50]. The YXXM motif found in the human CD96 cytoplasmic tails can be defined as a functional domain of DAP10 as well as SH2 structure. It is a highly conserved region with a TXXM motif similar to the CD28 fragment in the cytoplasm. Upon phosphorylation, the YXXM motif binds to the regulatory subunit p85 of phosphatidylinositol 3 kinase (PI3K) to initiate the downstream growth factor receptor-binding protein 2 (Grb2) [51,52]. The Grb2 molecule then binds to Shc and Sos proteins to form a Shc–Grb2–Sos complex, which activates Sos, and in turn binds to the Ras protein present on the cell membrane to further activate Sos, triggering a cascade of downstream signals [52]. Active NK cells release a number of cytokines, facilitating cancer cell death [52]. As human CD96 contains both ITIM motifs and YXXM motifs, it is unknown whether the regulation of NK cells is co-activated or co-repressed, and what are its roles in distinct cancers [38]. Although mouse CD96 contains only the ITIM motif, this does rule out the possibility that mouse CD96 may have inhibitory effects on NK cells. In fact, some studies have found that by blocking CD96 with antibodies improved tumor growth in mice, when combined with PD-1 blockers [53]. Further studies are required to reveal the relevant issues.

The effect of CD96 on NK cells is not only limited to promoting or inhibiting NK cell activation, but can also affect the adhesion and metastatic ability of NK cells. Further, studies show that binding of CD96 to CD155 can enhance the uptake of surface molecules within NK cells and target cells [42]. Although NK cells contain a co-stimulatory receptor like CD226 as well as a co-inhibitory receptor like TIGIT, whether these binding promotes or inhibits NK cell activation depends on the malignancy type.

### 6.2. CD96 Regulates CD8^+^ T Cells

CD96 regulation of CD8^+^ T cells has received significantly less attention than CD96 regulation of NK cells. CD96 was positively related to the number of CD8^+^ T cells in tumor-infiltrating tissues, suggesting that CD96 is most likely associated with CD8^+^ T cell dysfunction [38]. The use of mouse CD96 antibodies is shown to significantly inhibit the growth of colorectal cancer, melanoma, and subcutaneous fibrosarcoma in mice, a mechanism independent of NK cells and requires the involvement of CD8^+^ T cells and IFN-γ [28]. A combination of co-stimulatory and co-inhibitory receptors expressed on CD8^+^ T cells assist in the CD8^+^ T cell activity [54]. Using bioinformatic research tools, it was shown that CD96 expression was lower in rectal cancer, melanoma, and fibrosarcoma compared to paraneoplastic tissue, which implies that CD96 could promote CD8^+^ T cell activation as a co-stimulatory receptor. On the contrary, CD96 has also been shown to negatively regulate CD8+ T cells when combined with PD-1 [54]. Altogether, the majority of the findings support that CD96 functions as a TIGIT-like co-inhibitory receptor regulating CD8^+^ T cells. In terms of the role of CD96 as a co-stimulatory receptor regulating CD8^+^ T cells, it has been shown that CD96 cross-links with mouse or human CD8^+^ T cells, inducing activation and production of effector cytokines, and that CD96 can convert its activation signal via the MEK-ERK pathway [30]. Most published data demonstrate the role of CD96 in tumor cell metastasis mediated by NK cells rather than CD8^+^ T cells, necessitating research into CD96 regulation of CD8^+^ T cells [30].

## 7. CD96 as an Immune Regulator in Cancers

Based on the evidence described above, we can conclude that CD96 may serve as an immune check point molecule in cancer. Therefore, it is important to understand the various malignances in which this specific molecule may function. 

### 7.1. Liver Cancer

The interaction of co-inhibitory receptors CD96 and TIGIT, the co-stimulatory receptor CD226, and the co-ligand CD155 regulate the immune system’s response to tumors. CD96 is abundantly expressed on NK cells and in tumors. However, NK cell function is often impaired resulting in decreased IFN-γ production and reduced cytotoxicity. Studies have shown that IFN-γ production inversely correlates with the percentage of CD96^+^ NK cells in liver tissues and that reduced IFN-γ production could impair tumor immune responses. On the other hand, NK cell’s cytotoxicity against tumor cells expressing CD155 could be improved by disrupting the binding of CD96 to CD155 with a CD96 antibody. Compared to CD96-NK cells, CD96^+^ NK cells overexpressed the inhibition of relative molecules, such as PD-1, TIGIT, natural killer group 2A (NKG2A), and CD355, while downregulating activating relative molecules like CD226, CD69, killer cell lectin receptor G1 (KLRG1), and NKp30. CD96+ NK cells predominantly express transforming growth factor-β (TGF-β) and IL-10, while IL-15, perforin, T-bet, and granzyme B are less expressed. This suggest that the function of CD96^+^ NK cells tends to perform poorly. Moreover, CD96 can be used as a potential prognostic marker to predict survival in patients with hepatocellular carcinoma. The findings suggest that the higher the expression of CD96 in the tumor, the higher the deterioration and recurrence rates, and the shorter patient survival times [29]. A higher expression of the immune suppressive checkpoints CD96, PD-1, and TIGIT is seen in CD49a^+^ NK cells, with the number of CD49a^+^ NK cells positively corelated with the presence of malignant disease and a poor prognosis in patients with hepatocellular carcinoma [55]. Furthermore, a significant correlation between immune evasion and CD96 in hepatocellular carcinoma tissues with high NK cell infiltration has been demonstrated [56]. Bioinformatics-based analysis of CD96 gene expression in the tissues of patients with hepatocellular carcinoma revealed an increased expression of CD96, suggesting the potential role of CD96 in assisting tumor cells to evade the immune system [38]. 

Taken together, these findings suggest that CD96 can negatively regulate tumor immunity in hepatocellular carcinoma through lymphocytes such as CD8^+^ T cells and NK cells. However, very few studies go into details about the mechanisms by which CD96 regulates the functions of CD8^+^ T cells as well as NK cells and contributes to immunity in hepatocellular carcinoma.

### 7.2. Glioma

There has been less research done on the role of CD96 in glioma. Interestingly, two bioinformatics-based studies revealed that CD96 was significantly expressed in citrate dehydrogenase (IDH) wild-type and mesenchymal subtype gliomas and positively correlated to glioblastoma. The immune permeability testing results showed a strong correlation between CD96 and the infiltration levels of CD4^+^ T cells, CD8^+^ T cells, neutrophils, macrophages, and dendritic cells in low-grade gliomas and glioblastoma multiforme. In addition, CD96 was strongly associated with the immune suppressive receptors PD-1, TIGIT, CTAL-4, and TIM-3. Univariate and multivariate Cox analysis showed CD96 as an independent predictor in glioma [57,58].

### 7.3. Lung Cancer

CD96, a co-inhibitory molecule, and the co-stimulatory molecule CD226 bind CD155 competitively. An imbalance between these molecules may result in an increased or decreased immune response. Tumor-infiltrating lymphocytes (TILs) from the patients with lung adenocarcinoma (LUAD), for instance, express higher levels of CD96 and lower levels of CD226 than that in the peripheral circulating blood, which suggests an imbalance between CD96 and CD226 resulting in the decreased tumor immune response in LUAD. Expression of IFN-γ in CD8^+^ T cells was decreased when co-cultured with LUAD cells. On the contrary, blocking CD96 expression in this co-culture system increased IFN-γ production in CD8^+^ T cells. IFN-γ expression in CD8^+^ T cells was further reduced when co-cultured with LUAD cells overexpressing CD155. This suggests that increasing CD8^+^ T cell activity while targeting CD96/CD155 could be a potential therapeutic approach for treating LUAD patients [59,60].

### 7.4. Melanoma

Mice studies found that blocking CD96 can reduce lung metastasis of melanoma cells by controlling NK cell activity and IFN-γ production. Further, combining the blockade of CD96 and TIGIT could enhance the inhibitory effect on lung metastasis of melanoma cells [59,60].

### 7.5. Cervical Cancer

Patients with cervical cancer resistant to PD-1 blockade therapy showed considerably higher levels of CD96 expression in their CD8^+^ TILs. Compared to CD96^−^PD^−^1^+^CD8^+^ TILs, CD96^+^PD-1^+^CD8^+^ TILs exhibited an inherent terminally exhausted effector phenotype. As a result, blocking both CD96 and PD-1 enhanced CD8^+^ TILs’ anti-tumor activity [54].

### 7.6. AML

As leukemic stem cells (LSCs) cannot be effectively eradicated, it is challenging to completely cure acute myeloid leukemia (AML) in most patients using chemotherapy alone. We define CD34^+^CD38^−^ AML cells as LSCs, and FACS analysis showed that the majority (74 ± 25.3%) of LSCs expressed CD96, while only a minority (4.9 ± 1.6%) of normal hematopoietic stem cells (HSC) weakly expressed CD96. This finding holds true for pediatric AML as well [61]. To evaluate whether CD96^+^ AML cells are enriched for LSC activity, the researchers transplanted CD96^+^ and CD96^−^ cells AML cells into highly immunodeficient Rag2^−/−^γc^−/−^ mice. The transplanted mice were sacrificed 6–10 weeks later, and the findings showed that only CD96^+^ cells proliferated abundantly in four of the five mouse bone marrow samples. These findings suggest that CD96 is a cell surface marker on many AML-LSCs and could be a target for LSC-specific therapy [61,62,63]. Likewise, it was shown that AML patients had a higher percentage of CD96 than those with acute lymphoblastic leukemia (ALL), with the percentage of CD96 in each subgroup of AML: myeloblasts/promyelocites > granular myeloblasts > monoblasts [64]. Although the level of CD96 expression in T cells and NK cells was marginally higher in AML patients compared to healthy individuals, it was not statistically significant overall. Nonetheless, about half of T cells and NK cells in healthy individuals express CD96 [65]. This suggests that CD96 may aid in the prognosis of AML patients as well as target for immunotherapy.

## 8. Conclusions

Immune checkpoint inhibitors have revolutionized cancer care and markedly improved patient survival. Immune checkpoint inhibitors must, however, address problems such as severe treatment-related side effects and post-treatment drug resistance, as well as ways to improve the proportion of patients who respond to treatment. Combination therapy can be effective in improving patient response rates and reducing problems such as patient resistance. For instance, the combination of PD-1 and CTLA-4 did improve response rates in the patients receiving this therapy. As only a few immune checkpoint inhibitors are available for patient treatment, there is a need to accelerate the discovery of more effective immune checkpoints, such as CD96. The expression of CD96 in most cancer tissues is higher than in paracancerous and normal tissues. At the same time, the abundance of CD96 positively correlates with the infiltration level of immune cells in many cancers. Based on this evidence, we can propose that targeting CD96 in cancer may enhance the killing function of immune cells, thereby improving patient outcomes. Thus, CD96 may be a promising therapeutic target, especially in the context of some preclinical studies indicating that inhibition of the immune checkpoint CD96 has good efficacy and safety. There are a few relevant studies, limited to bioinformatics analysis, animal, and cell experiments only. However, there is still a long way to go before entering human clinical trials. Overall, CD96 appears to be a promising candidate for immune checkpoint blockade therapy.

## Figures and Tables

**Table 1:** Expression of CD96 in normal tissues and tumors.

Normal tissues	HPA database	High expression: T cell, NK cell
		Medium expression: lymph node, tonsil, urinary bladder, spleen, appendix, and B cells
		Low expression: small intestine and granulocytes
Tumor tissues	Oncomine database	High expression: brain cancer, breast cancer, kidney cancer, and leukemia
	TCGA database	High expression: adrenocortical cancer, breast invasive cancer, endocervical adenocarcinoma, cholangiocarcinoma, colon adenocarcinoma, esophageal carcinoma, glioblastoma, head and neck squamous cell carcinoma, kidney renal clear cell carcinoma, kidney renal papillary cell carcinoma, acute myeloid leukemia, lower grade glioma, liver hepatocellular carcinoma, ovarian serous cystadenocarcinoma, pancreatic adenocarcinoma, skin cutaneous melanoma, stomach adenocarcinoma, and testicular germ cell tumors
		Low expression: lung cancer, rectal cancer, and thyroid cancer

## Data Availability

No new data were created or analyzed in this study. Data sharing is not applicable to this article.
